# Enhanced Thermal and Dielectric Properties of Polyarylene Ether Nitrile Nanocomposites Incorporated with BN/TiO_2_-Based Hybrids for Flexible Dielectrics

**DOI:** 10.3390/polym15214279

**Published:** 2023-10-31

**Authors:** Yong You, Siyi Chen, Shuang Yang, Lianjun Li, Pan Wang

**Affiliations:** 1 Key Laboratory of General Chemistry of the National Ethnic Affairs Commission, School of Chemistry and Environment, Southwest Minzu University, Chengdu 610041, China; fishpiee@icloud.com (S.C.); polymerysnature@163.com (S.Y.); l61256676035@163.com (L.L.); 2 Key Laboratory of Pollution Control Chemistry and Environmental Functional Materials for Qinghai-Tibet Plateau of the National Ethnic Affairs Commission, School of Chemistry and Environment, Southwest Minzu University, Chengdu 610041, China; 3 School of Mechanical Engineering, Chengdu University, Chengdu 610106, China; wangpan@cdu.edu.cn

**Keywords:** polyarylene ether nitrile, nanohybrid, nanocomposites, thermal stability

## Abstract

Outstanding high-temperature resistance, thermal stability, and dielectric properties are fundamental for dielectric materials used in harsh environments. Herein, TiO_2_ nanoparticles are decorated on the surface of BN nanosheets by internal crosslinking between polydopamine (PDA) and polyethyleneimine (PEI), forming three-dimensional novel nanohybrids with a rough surface. Then, an ether nitrile (PEN) matrix is introduced into the polyarylene to form polymer-based nanocomposite dielectric films. Meanwhile, the structure and micromorphology of the newly prepared nanohybrids, as well as the dielectric and thermal properties of PEN nanocomposites, are investigated in detail. The results indicate that TiO_2_ nanoparticles tightly attach to the surface of BN, creating a new nanohybrid that significantly enhances the comprehensive performance of PEN nanocomposites. Specifically, compared to pure PEN, the nanocomposite film with a nanofiller content of 40 wt% exhibited an 8 °C improvement in the glass transition temperature (*T*_g_) and a 162% enhancement in the dielectric constant at 1 kHz. Moreover, the dielectric constant–temperature coefficient of the nanocomposite films remained below 5.1 × 10^−4^ °C^−1^ within the temperature range of 25–160 °C, demonstrating excellent thermal resistance. This work offers a method for preparing highly thermal-resistant dielectric nanocomposites suitable for application in elevated temperature environments.

## 1. Introduction

In recent years, as the 5G era unfolds, the electronics industry is swiftly moving towards miniaturization and high-speed advancements. This shift, accompanied by the diversification of electronic devices and the quest for smaller dimensions, imposes greater demands on circuit complexity and signal transmission speed [1]. As an important part of electronic devices, dielectric materials play a crucial role in them. In addition, dielectric constant and loss are one of the important indicators of dielectric materials. Among them, the dielectric constant is used to characterize the ability to bind charges. For the same breakdown strength and dielectric loss, a higher dielectric constant means a higher ability to bind charges and a higher ability to store energy. Dielectric loss refers to the dielectric generating a conductive current under the action of an applied electric field, consuming part of the electrical energy and converting it into thermal energy. The causes of dielectric loss include leakage loss, ionization loss, polarization loss, and structural loss. Therefore, polymer-based composites with a high dielectric constant and low dielectric loss are widely used in energy storage and flexible sensor devices because of their excellent dielectric properties and flexibility [2,3]. To tackle more rigorous application environments and explore capacitor usage at higher temperatures, researchers have developed novel polymer-based nanocomposites. With the advancement of society, various industries such as electric vehicles, oil extraction, and aerospace are increasingly requiring dielectric materials operating at high temperatures, but the low glass transition temperature (*T*_g_) of polymers often limits their application in this field. Fortunately, high-performance polymers such as polyphenylene sulfide [4], polyimide [5,6], and polyarylene ether nitrile (PEN) [7] have emerged. These high-performance polymers have high glass transition temperatures, enabling them to be applied at temperatures in excess of 150 °C [8,9,10]. Particularly, due to the unique multi-cyclic structure of PEN, it has a high glass transition temperature, excellent mechanical properties, good processing and forming characteristics, radiation protection and flame retardant properties, and it has been used in automotive and industrial production and other fields [11,12]. Furthermore, with the rigid benzene ring structure on the PEN backbone and the presence of nitrile (-CN) groups on the side chain, PEN exhibits excellent thermal stability and mechanical properties and improved dielectric properties. In addition, nitrile groups contribute to better adhesion between polymers and nanofillers. Therefore, it is precisely these excellent properties that have prompted researchers to use PEN as a matrix to develop composite films and investigate the effect of fillers on their comprehensive properties.

To enhance the dielectric constant of polymer nanocomposites, it is common practice to incorporate ceramic or conductive fillers into the polymer matrix. Usually, polymer nanocomposites filled with conductive fillers (carbon nanotubes, graphene, and carbon fibers) can achieve high dielectric constants and exhibit good mechanical properties at relatively low volume fractions [13,14,15]. However, according to percolation theory, polymer matrix composites undergo a transition from insulators to conductors, thus inevitably increasing the dielectric loss [16]. In contrast, polymer nanocomposites equipped with ceramic fillers such as BaTiO_3_, SrTiO_3_, and TiO_2_ tend to have a high dielectric constant while keeping their dielectric losses at a very low level [17,18,19].

In addition, in order to further improve the thermal conductivity of PEN, it is essential to fill it with high thermal conductivity inorganic nanomaterials (such as carbon nanotubes (CNTs), graphite (GO) and boron nitride (BN), etc.) [20,21,22]. Among them, hexagonal boron nitride, characterized by a graphite-like layered structure, stands out as an exceptional insulating material with an ultra-wide bandgap. Two-dimensional BN nanosheets, derived from bulk hexagonal boron nitride (h-BN) through exfoliation, show great promise in electronic packaging and high-power devices due to their high thermal conductivity, outstanding thermal and chemical stability, and ultra-wide bandgap [22]. Li et al. [23] incorporated boron nitride nanosheets into siloxane resin to create crosslinked nanocomposites, c-BCB/BNNS. The resulting c-BCB/BNNS displayed a glass transition temperature surpassing 350 °C, thereby confirming its applicability as a dielectric material in temperatures up to 300 °C. Although boron nitride has excellent thermal conductivity, significant thermal stability, and electrical insulation [24], the dispersion of nanofillers within the polymer matrix is often compromised, resulting in significant aggregation and high dielectric losses [25,26,27]. Furthermore, the prepared composites exhibit poor interfacial and mechanical properties without surface treatment. Consequently, a simple and efficient method for surface modification of nanofillers is required to fully exploit the exceptional properties of boron nitride [28].

Recent research has unveiled the strong adhesive properties of dopamine (DA), and its abundant catechol groups make it suitable for surface modification on various materials. Under alkaline conditions, DA can undergo self-polymerization, forming polydopamine (PDA) organic layers on different substrate surfaces. However, achieving a uniform and stable PDA layer on material surfaces is challenging. In this work, to address this issue, we adopted a co-modification method that combines PDA with polyethyleneimine (PEI), and developed a novel three-dimensionally structured nanoparticle (BN-TiO_2_)@(PDA + PEI) through the covalent interaction between PDA and PEI, in which TiO_2_ nanoparticles were loaded onto BN nanosheets. This composite possesses abundant polar groups (-NH_2_ and -OH) on its surface, significantly improving the interfacial compatibility with the polymer matrix. Utilizing continuous ultrasound, various mass fractions of (BN-TiO_2_)@(PDA + PEI) were incorporated into the PEN matrix, and PEN nanocomposite films were prepared using the cast film-forming method. This approach ensures good dispersion and compatibility, consequently enhancing the comprehensive performance of the PEN nanocomposite films as dielectric materials.

## 2. Experimental Methods

### 2.1. Materials

Boron nitride (BN, diameter: 1–5 µm, thickness: ~5 nm, >99%), titanium dioxide (TiO_2_, anatase, ~25 nm, 99.8%) were provided from Shanghai Titan Technology Co., Ltd. (Shanghai, China). Tris (hydroxymethyl) aminomethane (99.9%), dopamine hydrochloride (DA, 98%) were supplied by Chengdu Shuobo Research and Innovation Technology Co., Ltd. (Chengdu, China). N-methyl-2-pyrrolidone (NMP, 98%), hydrochloric acid (HCl, AR), sodium hydroxide (NaOH, AR), and polyethyleneimine (PEI, AR) were purchased from Chengdu Kelong Reagent Co., Ltd. (Chengdu, China). All materials were used without further purification. 

### 2.2. Preparation of (BN-TiO_2_)@(PDA + PEI) Nanohybrids

**Figure 1:**
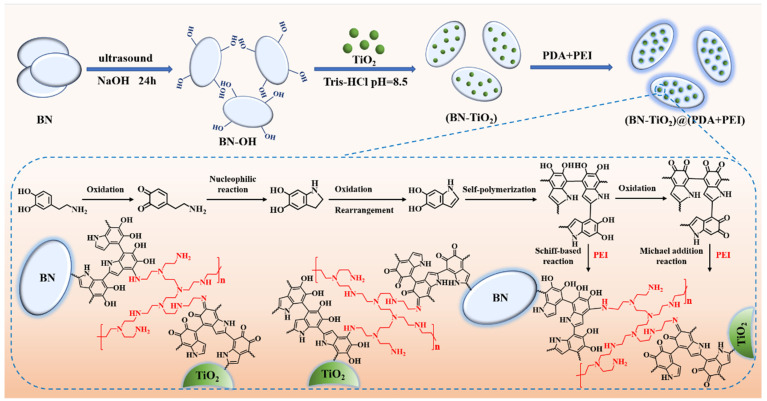
Schematic diagram of the preparation of (BN-TiO_2_)@(PDA + PEI) nanohybrids.

The three-dimensionally structured BN-TiO_2_ nanohybrids were prepared by DA in situ using self-polymerization and its internal crosslinking with PEI ((BN-TiO_2_)@(PDA + PEI)), and the corresponding preparation process is shown in Figure 1. Firstly, 8 g BN nanosheets were added to a three-necked bottle containing 100 mL of NaOH solution, followed by continuous stirring and ultrasound for 4 h to disperse and peel off. Then, after continuing to stir for another 20 h, the hydroxylated BN (BN-OH) solid product was obtained by vacuum filtration. Immediately afterwards, the BN-OH nanosheets were washed to neutral using deionized water and dried at 60 °C for 12 h.

After that, 0.4 g BN-OH and 0.2 g TiO_2_ were added to a three-necked bottle containing 148 mL of Tris-HCl buffer solution with pH = 8.5 and then stirred with ultrasound for 30 min. Subsequently, 0.3 g of dopamine hydrochloride was dissolved in 1 mL of UP water and slowly added to the mixture. After ultrasonic stirring for 2 h, 0.9 mL of polyethyleneimine was added and the reaction continued for 4 h. Next, the solution was filtered, and the product was washed to neutral with ethanol and UP water, respectively. Finally, the product was dried in a 60 °C oven for 12 h to obtain (BN-TiO_2_)@(PDA + PEI) nanohybrids.

### 2.3. Preparation of PEN Nanocomposite Films

PEN was synthesized according to the literature [25]. PEN/(BN-TiO_2_)@(PDA + PEI) nanocomposite film was prepared by solution casting method, and the detailed operation process is as follows: Firstly, a certain amount of (BN-TiO_2_)@(PDA + PEI) nanohybrids was added to 20 mL NMP, followed by stirring and sonication for 1 h. Then, the flask was transferred to a heating sleeve and metered PEN powder was added. After the PEN was completely dissolved, it was poured on a flat glass plate in the oven and heated by a procedure: 80 °C, 100 °C, 120 °C for 1 h, and 160 °C, 200 °C for 2 h, respectively. After the heating process, PEN/(BN-TiO_2_)@(PDA + PEI) nanocomposite film was obtained. Different proportions of nanocomposite films (0, 10, 20, 30, 40 wt%) were prepared by changing the feeding ratio, which were named as follows: PEN, PEN/(BN-TiO_2_)@(PDA + PEI) 10, PEN/(BN-TiO_2_)@(PDA + PEI) 20, PEN/(BN-TiO_2_)@(PDA + PEI) 30, PEN/(BN-TiO_2_)@(PDA + PEI) 40. At the same time, PEN nanocomposite films with the (BN-TiO_2_) content of 10 wt% to 40 wt% were prepared as a comparison, where BN:TiO_2_ = 2:1. The thickness of all nanocomposite films is approximately 50 µm.

### 2.4. Characterization

The chemical structure of nanohybrids was characterized by X-ray photoelectron spectroscopy (XPS, Thermo Scientific ESCALAB 250Xi, Waltham, MA, USA) and Fourier-transform infrared (FTIR, Thermo Nicolet, IR 200, Waltham, MA, USA) spectra from 400–4000 cm^−1^. The microscopic morphology of nanohybrids was observed by transmission electron microscopy (TEM, JEOL JEM-f20, Tokyo, Japan) and scanning electron microscopy (SEM, JEOL JSM-7500LV, Tokyo, Japan). The X-ray diffraction (XRD, Beijing Pu-Analysis General Instrument, Beijing, China, XD-6, 4°/min) analysis was performed with a testing range of 5° to 85°. Thermal gravimetric analysis (TGA, TA, Q50, New Castle, DE, USA, 20 °C/min) was conducted to characterize the thermal properties of nanohybrids in a N_2_ atmosphere from 50–800 °C. The zeta potential was tested using a zetasizer instrument (Nano zs, Malvern, UK) at room temperature. Mechanical properties analyses of PEN nanocomposites were carried out on an electromechanical universal testing machine (QX-W200, Shanghai Qixiang Testing Instrument Co., Ltd., Shanghai, China). Differential scanning calorimetry (DSC, TA, Q2000, New Castle, DE, USA, 10 °C/min) with a temperature range of 50–400 °C was used to determine the thermal properties of PEN nanocomposites. All samples were heated in a first cycle to remove thermal history and then naturally cooled to room temperature, followed by a second heating to a set temperature. The heat transfer properties were tested using an infrared thermal imager (E40, FLIR Systems Inc, NASDAQ: FLIR, Wilsonville, OR, USA). Dielectric properties (dielectric constant and loss) were measured by an LCR meter (TH2819A, Tonghui Electronics Co., Ltd., Dongguan, China) with a frequency of 100 Hz-1 MHz at an alternating voltage (AC) of 1.0 V. All the samples were cut into 1 × 1 cm^2^ rectangular squares and coated with conductive silver paste on both sides to form a capacitor. The dielectric properties–temperature relationship of the nanocomposites was tested in the temperature range from 25 °C to 250 °C at a frequency of 1 kHz. The water absorption of nanocomposites was calculated by the formula (W_2_ − W_1_)/W_1_ × 100%, where W_1_ is the weight of each sample before placing it in deionized water, and W_2_ is the weight of each sample after immersing it in deionized water for 24 h.

## 3. Results and Discussion

### 3.1. Characterization of (BN-TiO_2_)@(PDA + PEI) Nanohybrids

**Figure 2:**
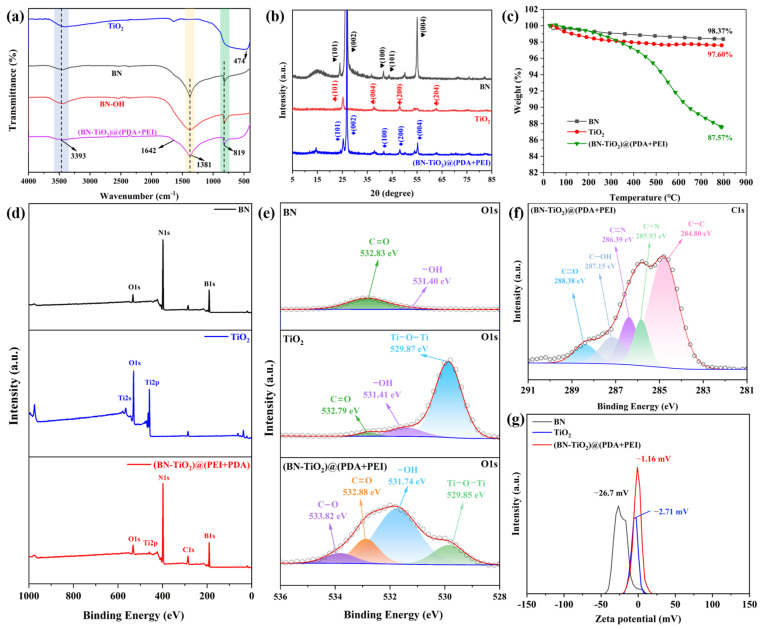
Characterization of nanohybrids: (**a**) FTIR spectra; (**b**) XRD patterns; (**c**) TGA curves; (**d**) XPS survey spectra; (**e**) O1s, (**f**) C1s XPS spectra of nanohybrids; (**g**) zeta potential spectra.

The chemical composition and crystal structure of the nanohybrids are characterized in detail, and are shown in Figure 2. Figure 2a shows the FTIR spectra of TiO_2_, BN, BN-OH, and (BN-TiO_2_)@(PDA + PEI), respectively. The figure reveals a broad absorption band around 474 cm^−1^, corresponding to the frame vibration of Ti-O in TiO_2_ [29,30]. Additionally, the absorption peak at 3393 cm^−1^ corresponds to the O-H and N-H absorption peaks, associated with the amino group (-NH_2_) in PDA and PEI. Furthermore, the absorption peaks of BN at 1381 cm^−1^ and 819 cm^−1^ represent the telescopic vibration of the B-N bond and the bending vibration of the B-N-B bond, respectively [31]. The absorption peak at 1642 cm^−1^ corresponds to the stretching vibration of the C=C bond in the benzene ring of PDA. Based on the above analysis, the FTIR spectra of (BN-TiO_2_)@(PDA + PEI) exhibit characteristic absorption peaks of the aforementioned substances, confirming the successful modification of BN-TiO_2_ by the PDA and PEI layers.

Figure 2b illustrates the XRD patterns of the nanohybrids obtained with an X-ray diffractometer. Clear characteristic diffraction peaks can be observed for BN and (BN-TiO_2_)@(PDA + PEI) at 2θ = 26.76°, 41.60°, 43.87°, and 55.16°, corresponding to the crystal planes of BN (002), (100), (101), and (004) (JCPDS card No. 45-0893) [32]. Importantly, the (BN-TiO_2_)@(PDA + PEI) nanohybrid exhibits strong diffraction peaks at 2θ = 25.31°, 37.91°, 48.01°, 53.9°, and 62.7°, corresponding to the crystal planes of TiO_2_ (101), (004), (200), (105), and (204) (JCPDS card No. 21-1272) [33]. Hence, the XRD pattern of (BN-TiO_2_)@(PDA + PEI) confirms the presence of characteristic diffraction peaks for both BN and TiO_2_, indicating the successful loading of TiO_2_ onto the surface of BN. 

Thermal stability under a N_2_ atmosphere can be analyzed using TGA curves of nanohybrids. As shown in Figure 2c, all three nanoparticles display varying degrees of mass loss with increasing temperature. Pure BN and TiO_2_ exhibit mass losses of 1.64% and 2.40% in the range of 50–800 °C, respectively. It is mainly attributed to the presence of limited hydroxyl groups on their surfaces, indicative of excellent thermal stability. However, (BN-TiO_2_)@(PDA + PEI) experiences significant thermal decomposition above 350 °C, with a mass loss of 10.78% at 800 °C, which is mainly attributed to the decomposition of the PDA and PEI modification layers on the surface of (BN-TiO_2_). These results further confirm the successful coating of PDA and PEI on the surfaces of BN and TiO_2_.

As can be seen from Figure 2d, two strong peaks at about 191 eV and 398 eV are observed in the full spectrum of BN, which belong to B1s and N1s, respectively. Furthermore, there are three distinct peaks at around 565 eV, 531 eV, and 459 eV in the spectrum of pure TiO_2_, corresponding to Ti2s, O1s, and Ti2p, respectively. In addition, the characteristic diffraction peaks of Ti2p and B1s in the full spectrum of (BN-TiO_2_)@(PDA + PEI) confirm the presence of BN and TiO_2_ in the (BN-TiO_2_)@(PDA + PEI) nanohybrids. At the same time, an obvious diffraction peak at 287 eV appeared in the full spectrum of (BN-TiO_2_)@(PDA + PEI), which is assigned to the C1s coming from the PDA and PEI. In addition, in order to further verify the existence of the organic modification layer PDA + PEI on the surface of (BN-TiO_2_)@(PDA + PEI), the O1s spectrum of the BN, TiO_2,_ and (BN-TiO_2_)@(PDA + PEI) are also illustrated in Figure 2e. It is clear that there are two weak peaks at 531.40 eV (-OH) and 532.83 eV (C=O) in the O1s spectrum of BN, which is probably assigned to a few contents of -OH groups and the adsorbed CO_2_ on the surface of BN, respectively. The peaks of -OH and C=O bonds also appear at the same location in the O1s spectrum of the TiO_2_. Comparing (BN-TiO_2_)@(PDA + PEI) with BN and TiO_2_, the O1s spectrum of (BN-TiO_2_)@(PDA + PEI) can be quantitatively differentiated into four distinct peaks at 529.85 eV, 531.74 eV, 532.88 eV, and 533.82 eV, corresponding to Ti-O-Ti, -OH, C=O, and C-O bonds, respectively. Among them, the -OH, C=O, and C-O bonds mainly come from the (PDA + PEI) organic modification layer of (BN-TiO_2_)@(PDA + PEI) [34,35]. Furthermore, Figure 2f displays the C1s spectrum of (BN-TiO_2_)@(PDA + PEI), which exhibits five distinct diffraction peaks at 284.80 eV, 285.93 eV, 286.39 eV, 287.15 eV, and 288.38 eV. These peaks correspond to the C-C, C-N, C=N, C-OH, and C=O bonds in the modified layer [36], respectively, indicating further chemical reactions between PDA and PEI. These results provide evidence that the surface of the nanohybrids has been effectively capped with the (PDA + PEI) organic layer. Figure 2g shows the zeta potential pattern of the nanohybrids. It is evident from the figure that all nanoparticles exhibit negative zeta potentials, specifically −26.7 mV, −2.71 mV, and −1.16 mV, respectively. This negative charge can be attributed to the presence of hydroxyl groups on the surface of BN and TiO_2_, which weakens their ability to acquire protons compared to water. Additionally, the presence of PDA and PEI layers on the surface of the (BN-TiO_2_)@(PDA + PEI) nanohybrids introduces amino groups from PEI, resulting in a reduction in the absolute value of the zeta potential.

**Figure 3:**
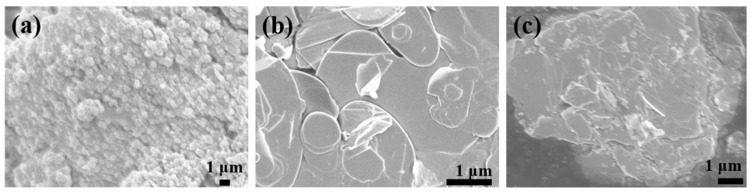
SEM images of functional nanohybrids: (**a**) TiO_2_; (**b**) BN; (**c**) (HNTs-TiO_2_)@(PDA + PEI).

For visual observation of the modification on the BN surface, scanning electron microscopy was employed, and the micromorphology of the modified BN is presented in Figure 3. Figure 3a illustrates the SEM image of pure TiO_2_, characterized by a grainy and smooth surface. Figure 3b displays the unmodified BN nanosheets, exhibiting a distinct layered structure with a flat and smooth surface. In Figure 3c, the SEM image of (BN-TiO_2_)@(PDA + PEI) nanohybrids is shown. The modification of BN results in a roughened surface, and the spherical TiO_2_ nanoparticles are tightly attached to the BN surface, forming a three-dimensional structure. This observation suggests that TiO_2_ nanoparticles are loaded onto the BN nanosheets through the bridging effect of PDA and PEI.

**Figure 4:**
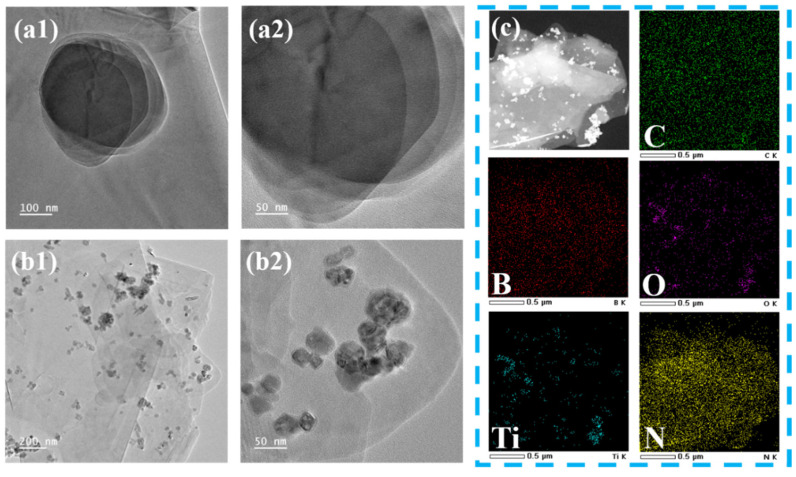
TEM images of functional nanohybrids: (**a1**,**a2**) BN; (**b1**,**b2**) (BN-TiO_2_)@(PDA + PEI) and (**c**) TEM mapping images of (BN-TiO_2_)@(PDA + PEI).

To further investigate the microscopic morphology of (BN-TiO_2_)@(PDA + PEI), TEM and EDX analyses were conducted. Figure 4(a1,a2) shows the TEM image, revealing the typical two-dimensional lamellar structure of BN with a smooth surface. However, Figure 4(b1,b2) demonstrates that TiO_2_ nanoparticles are loaded onto the surface of the BN nanosheets, and both surfaces are enveloped by an amorphous organic layer, resulting in a unique three-dimensional structure. This indicates that the surface of TiO_2_ nanoparticles and BN nanosheets are wrapped by the PDA layer through strong van der Waals forces and π-π interactions, and further loaded with the PEI layer through internal crosslinking interactions between PEI and PDA. Figure 4c presents the TEM mapping images of the nanohybrids, where the distribution of Ti and O elements on the nanosheet layer is clearly observed, confirming the successful loading of TiO_2_ onto the surface of BN nanosheets. Additionally, the C element fully covers the nanohybrids, providing further evidence that the surface of (BN-TiO_2_) is encapsulated by the PDA + PEI organic layer.

### 3.2. The Properties of Nanocomposites

**Figure 5:**
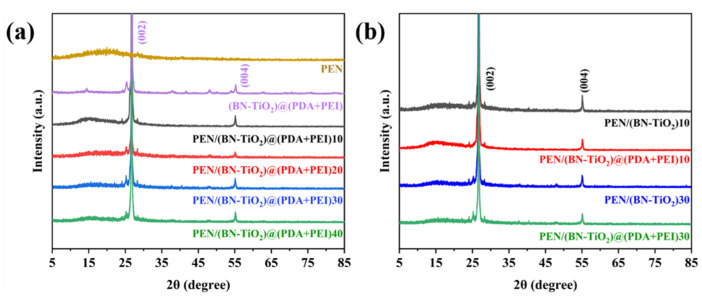
XRD patterns of nanocomposite films with (**a**) different nanofillers content and (**b**) the nanofillers before and after modification.

To examine the crystal structure of (BN-TiO_2_)@(PDA + PEI) nanohybrids in a PEN matrix, the XRD analysis was performed on nanocomposites filled with different nanofiller contents before and after modification. Figure 5a shows the XRD pattern, where the (002) and (004) crystal planes of boron nitride are observed at 2θ = 26.76° and 55.16°, respectively, while the diffraction peak at 2θ of 25.31° corresponds to the (101) crystal plane of titanium dioxide [32,33]. Moreover, the XRD pattern clearly displays the diffraction peaks of the PEN matrix, indicating that the modified BN and TiO_2_ effectively function in the PEN matrix. The intensity of the diffraction peaks increases with an increase in the nanofiller content, indicating the successful preparation of nanocomposite films with different filler ratios. 

Figure 5b presents a comparison of the XRD spectra between nanocomposite films containing 10 wt% and 30 wt% of (BN-TiO_2_) and (BN-TiO_2_)@(PDA + PEI) nanofillers. The characteristic diffracted peaks of BN and TiO_2_ can be observed at the corresponding diffraction angles, suggesting that the crystals of BN and TiO_2_ remain stable during the preparation process of the films at high temperatures. This result indicates the potential application of these materials as dielectric materials in harsh environments.

**Figure 6:**
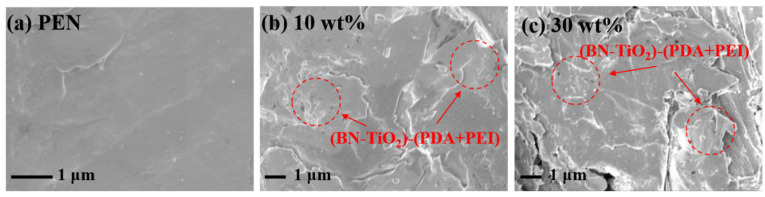
SEM images of PEN nanocomposite films: (**a**) PEN; (**b**) 10 wt%; (**c**) 30 wt%.

The interfacial compatibility between the nanohybrids and the polymer, as well as the dispersion of the nanohybrids in the polymer matrix, significantly influence the comprehensive performance of nanocomposites [37]. Therefore, the cross-section morphology of the nanocomposite films was examined using SEM, and the results are presented in Figure 6. In Figure 6a, the cross-section of the PEN matrix appears relatively smooth without any noticeable presence of two phases. Figure 6b displays the cross-section morphology of the nanocomposite dielectric films with a filler content of 10 wt% (BN-TiO_2_)@(PDA + PEI). It can be observed that the (BN-TiO_2_)@(PDA + PEI) nanohybrids exhibit good compatibility with the PEN matrix, as they are tightly encapsulated by the resin without any prominent bare leakage or phase separation. This desirable compatibility can be attributed to the presence of polar groups, such as hydroxyl (-OH) and amino (-NH_2_) groups, on the surface of the modified layer. These groups can form hydrogen bonding interactions with the nitrile group (-CN) on the molecular chain of the PEN matrix, thereby enhancing the compatibility between the fillers and the matrix [38]. However, upon further increasing the nanohybrid content, some defects like voids become evident on the surface of the nanocomposite films (Figure 6c). This occurrence is primarily due to the inevitable partial agglomeration of a large number of inorganic nanohybrids within the PEN matrix. In summary, the compatibility of the nanofillers with the PEN matrix is improved after the co-modification with PDA and PEI, thus providing favorable conditions for the preparation of high-performance nanocomposite films.

**Figure 7:**
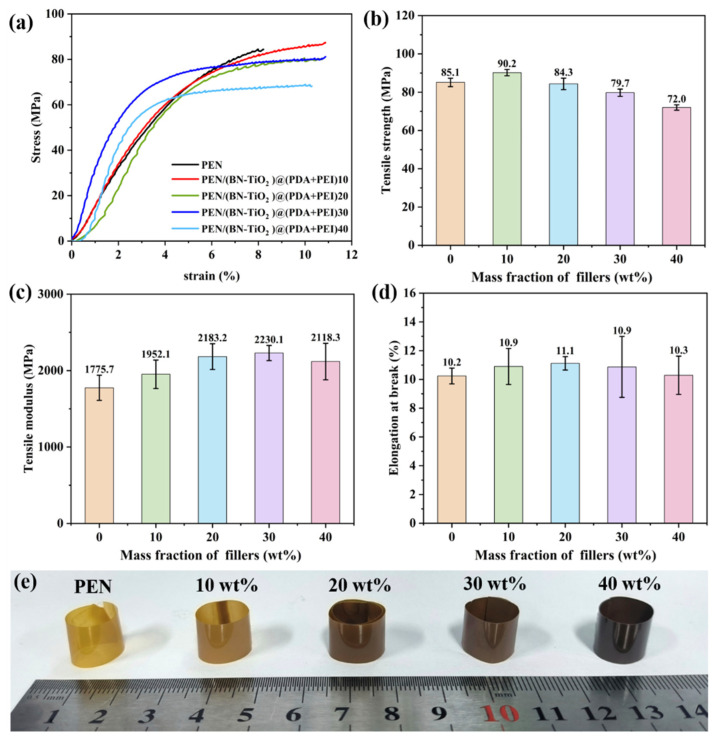
Mechanical properties of PEN nanocomposite films with different nanofillers: (**a**) stress–strain curves; (**b**) tensile strength, (**c**) tensile modulus, (**d**) elongation at break, and (**e**) physical photograph of nanocomposite films.

The mechanical properties of PEN nanocomposites were investigated through stress–strain testing of the nanocomposite film, as shown in Figure 7. Figure 7a displays the stress–strain curves of the PEN/(BN-TiO_2_)@(PDA + PEI) nanocomposite film, while the calculated tensile strength is illustrated in Figure 7b. It can be observed that the tensile strength of the nanocomposite films follows a general increasing trend until reaching a peak and subsequently decreasing. The pure PEN film exhibits a tensile strength of 85.1 MPa, while the PEN nanocomposite film with a 10 wt% filler content reaches a maximum tensile strength of 90.2 MPa, which is 5.99% higher than that of pure PEN. However, as the filler content further increases, the tensile strength gradually decreases. This behavior can be attributed to the formation of a three-dimensional nanohybrid structure with a rough surface due to the attachment of TiO_2_ onto the surface of the two-dimensional BN nanosheets. This structure increases the physical entanglement between the nanohybrids and the PEN molecular chains, forming physical crosslinking points in the nanocomposites at lower filler contents. These factors hinder the movement of the polymer molecular chains, thereby enhancing the tensile strength of the nanocomposites [39]. Additionally, the amino and hydroxyl groups in the organic modification layer (PDA + PEI) on the surface of the inorganic nanohybrids can form hydrogen bonds with the nitrile groups in the PEN matrix, thereby imparting some extent of chain mobility under external forces [38]. However, as the filler content increases further, partial agglomeration of the nanofillers within the PEN matrix occurs, resulting in defects and a reduction in the tensile strength of the nanocomposite film. This observation aligns with the findings from the SEM images. Nevertheless, it is noteworthy that even with decreased tensile strength, the nanocomposite film still maintains a tensile strength above 72.0 MPa at a 40 wt% filler content, which is considered a relatively high level. 

In addition, it can be seen from Figure 7c that with the increase in nanofiller content, the tensile modulus of nanocomposite films shows a trend of first increasing and then decreasing. This is mainly due to the introduction of inorganic rigid nanofillers to further enhance the rigidity of the nanocomposites, thereby increasing the tensile modulus of composite films to a certain extent. However, when the filling content is too high, local agglomeration of inorganic nanofillers inevitably occurs in the PEN matrix, which affects the tensile modulus of the nanocomposite. Furthermore, As shown in Figure 7d, the elongation at break for all nanocomposite films exceeds 10%, indicating good flexibility. To showcase the flexibility of the nanocomposite film further, all nanocomposite films are curled into cylindrical shapes, as demonstrated in Figure 7e. It is evident from the figure that the nanocomposite films can flexibly bend with arbitrary large curvatures, pointing towards potential applications in the field of flexible electronic materials.

**Figure 8:**
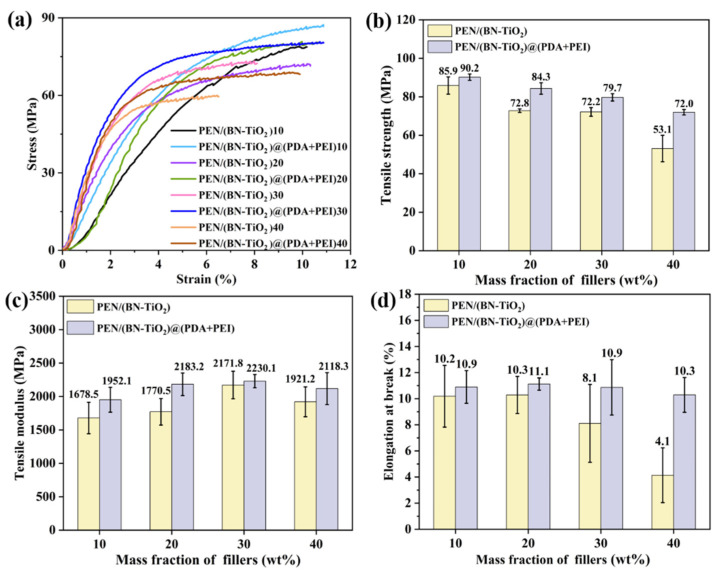
Mechanical properties of PEN nanocomposite films with different nanofillers: (**a**) stress–strain curves; (**b**) tensile strength; (**c**) tensile modulus; and (**d**) elongation at break.

To further explore the effect of interfacial modification of nanohybrids on the mechanical properties of nanocomposites, Figure 8 presents a mechanical comparison between PEN/(BN-TiO_2_) and PEN/(BN-TiO_2_)@(PDA + PEI) nanocomposite films with filler contents of 10 wt% to 40 wt%. Figure 8a shows the stress–strain curves of the PEN-based nanocomposite film, while the calculated tensile strength, tensile modulus, and elongation at break are illustrated in Figure 8b–d, respectively. It can be observed that the mechanical properties of PEN nanocomposite films containing (BN-TiO_2_) are lower compared to those with (BN-TiO_2_)@(PDA + PEI), indicating that the modified nanohybrids enhance the mechanical properties of PEN nanocomposites. This improvement can be attributed to the agglomeration phenomenon of unmodified BN and TiO_2_, resulting in non-uniform dispersion within the PEN matrix. In contrast, the presence of an organic modification layer on the surface of (BN-TiO_2_)@(PDA + PEI) effectively hinders the agglomeration of nanohybrids. Consequently, the modified nanohybrids exhibit better dispersion within the PEN matrix, leading to improved mechanical properties.

**Figure 9:**
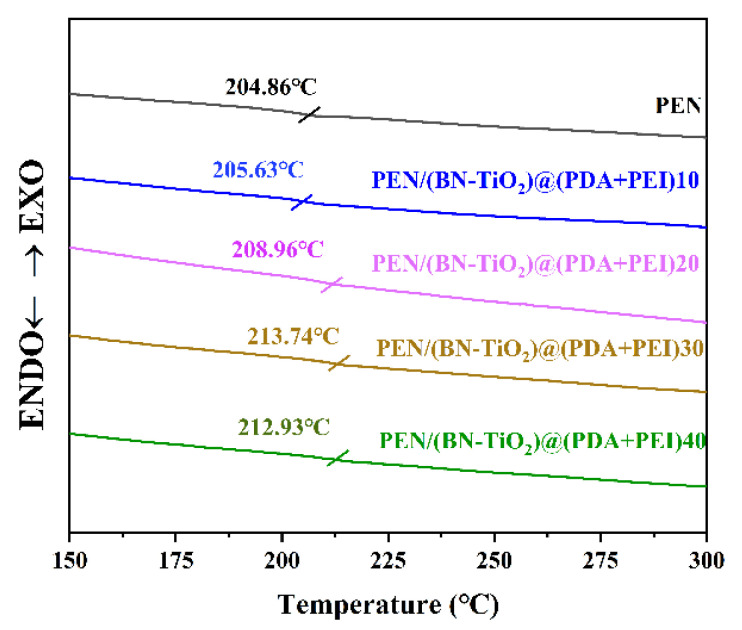
The DSC curves of nanocomposite films.

PEN is a kind of thermoplastic polymer material with good thermal properties, and glass transition temperature is the theoretical working temperature that affects the practical application of this type of material, so it is of great significance to explore its *T*_g_. The DSC curves of the PEN/BN-TiO_2_)@(PDA + PEI) nanocomposites are shown in Figure 9. 

It is clear that the *T*_g_ of pure PEN film is 204.86 °C, and the PEN nanocomposite films with various filler contents exhibit higher *T*_g_ values compared to pure PEN, which are 205.63, 208.96, 213.74, and 212.93 °C, respectively. The increase in *T*_g_ can be primarily attributed to the formation of physical crosslinking points between the (BN-TiO_2_)@(PDA + PEI) nanohybrids dispersed in the PEN matrix, which restricts the movement of PEN molecular segments. Additionally, the three-dimensional rough interface of the (BN-TiO_2_)@(PDA + PEI) nanohybrids enhances the physical entanglement between the nanofiller and the PEN molecular chains, further impeding the movement of the molecular chains to some extent [40].

**Figure 10:**
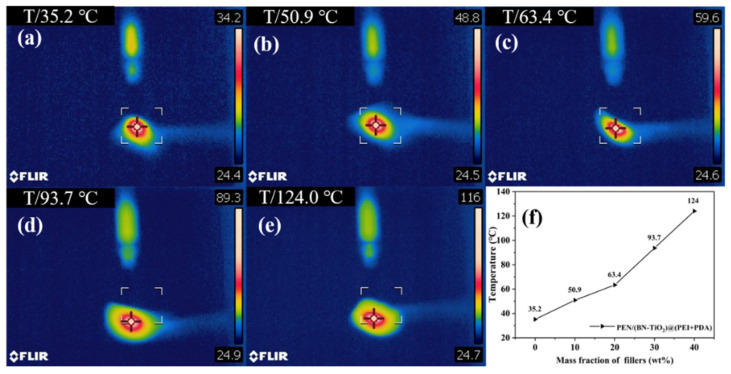
Infrared thermographic image of nanocomposite films: (**a**) PEN; (**b**) 10 wt%; (**c**) 20 wt%; (**d**) 30 wt%; (**e**) 40 wt%; and (**f**) the center temperature of nanocomposites.

Figure 10 illustrates the center temperatures of PEN nanocomposite films with different (BN-TiO_2_)@(PDA + PEI) nanofiller contents after 10 s of laser irradiation. It is evident that the center temperature of PEN/(BN-TiO_2_)@(PDA + PEI) nanocomposite films gradually increases with an increase in nanofiller content. The center temperature of the pure PEN film is 35.2 °C (Figure 10a), indicating weak thermal conductivity. 

However, as the filler content increases from 10 wt% to 40 wt% (Figure 10b–e), the center temperature of the nanocomposite films increases from 50.9 to 124.0 °C, respectively. This phenomenon can be attributed to the excellent thermal conductivity of BN, which is a two-dimensional nanosheet that effectively improves thermal conductivity when uniformly dispersed in the PEN matrix. Additionally, the presence of an organic modification layer on the surface of the nanohybrids enhances the interface compatibility between the nanofillers and the PEN matrix, thereby facilitating heat transfer through the organic–inorganic interface. These results demonstrate that this type of nanocomposite film efficiently transfers the heat generated during the operation of electronic devices, thereby improving the stability of electronic device operation [41].

**Figure 11:**
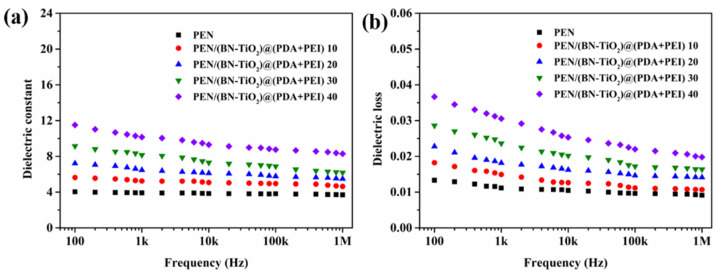
Dielectric properties of nanocomposite films: (**a**) dielectric constant; (**b**) dielectric loss.

Figure 11 shows the dielectric properties of PEN/(BN-TiO_2_)@(PDA + PEI) nanocomposite films from 100 Hz to 1 MHz at room temperature. In Figure 11a, it is evident that the permittivity of all nanocomposite films decreases with increasing frequency, while still exhibiting good permittivity–frequency stability overall. Moreover, the dielectric constant of the pure PEN film at 1 kHz is only 3.9. However, as the nanohybrid content increases from 10 to 40 wt%, the dielectric constant of the nanocomposite films at 1 kHz becomes 5.2, 6.5, 8.1, and 10.2, respectively. These results demonstrate that PEN/(BN-TiO_2_)@(PDA + PEI) 40 exhibits a 162% increase in dielectric constant compared to pure PEN. The increase in dielectric constant can be primarily attributed to two reasons: (1) The inorganic BN and TiO_2_ possess relatively high dielectric constants, effectively increasing the dielectric constant of the nanocomposite films when dispersed within the PEN matrix [25]. (2) The uniformly dispersed inorganic nanohybrids within the PEN matrix can be considered to be forming a micro-capacitor network. With an increase in nanofiller content, the number of micro-capacitors also increases, thereby elevating the dielectric constant of the system [34].

In addition, Figure 11b displays the dielectric loss of the PEN/(BN-TiO_2_)@(PDA + PEI) nanocomposite film. Although the dielectric loss of the nanocomposite films gradually increases with increasing filler content, it remains below 0.032 at 1 kHz for all nanocomposites. This is primarily attributed to the good compatibility between the interfacially modified (BN-TiO_2_)@(PDA + PEI) and the PEN matrix, which reduces the occurrence of defects and minimizes local charge aggregation at defects. Additionally, the hydrogen bonds formed between the amino and hydroxyl groups in the modified layer on the surface of the nanohybrids and the nitrile groups in the PEN molecular chain further reduce the interfacial polarization of the system [34]. As a result, the dielectric loss of the nanocomposite films is maintained at a relatively low level.

**Figure 12:**
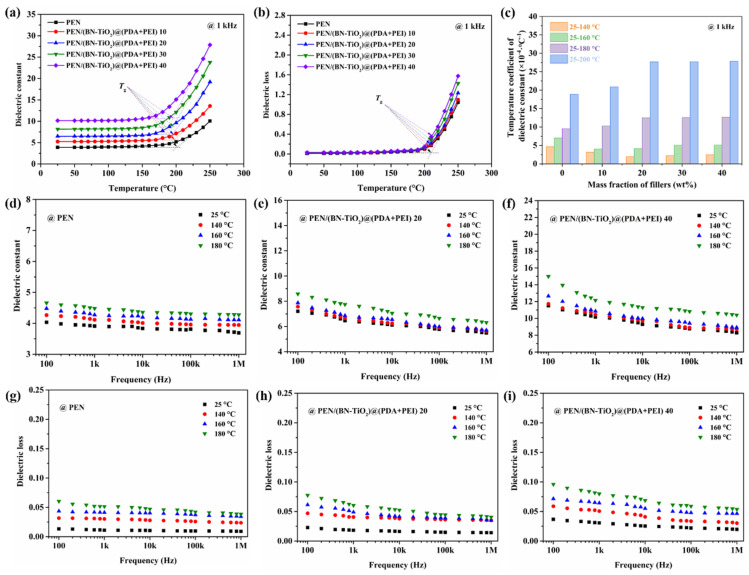
Dielectric properties of nanocomposite films: (**a**) dielectric constant–temperature relationship; (**b**) dielectric loss–temperature relationship; (**c**) dielectric constant–temperature coefficient; (**d**–**f**) dielectric constant of nanocomposites with changing frequency at different temperatures; (**g**–**i**) dielectric loss of nanocomposites with changing frequency at different temperatures.

To further investigate the correlation between dielectric properties and temperature, Figure 12 displays the relationship between dielectric properties and temperature. It is evident from Figure 12a that the dielectric constant (1 kHz) of nanocomposite films with 0 to 40 wt% nanofillers remains relatively stable below their respective *T*_g_. However, once the *T*_g_ of the nanocomposites is exceeded, the dielectric constant exhibits a significant increase. This phenomenon can be attributed to the restriction of molecular chain movement in the PEN nanocomposites at temperatures below its *T*_g_. Conversely, when the temperature is higher than *T*_g_, the PEN molecular chain begins to move at high temperature, resulting in increased internal polarization and a sharp rise in the dielectric constant [42]. Meanwhile, the dielectric loss–temperature relationship of PEN nanocomposites with 0 to 40 wt% nanofillers shows a similar change trend to that of the dielectric constant, which is shown in Figure 12b.

Furthermore, Figure 12c illustrates the dielectric performance–temperature relationship of all the nanocomposites, calculated using a formula reported in the literature [43]. Notably, within the temperature range of 25 to 160 °C, the dielectric constant–temperature coefficient of the nanocomposites remains below 5.1 × 10^−4^ °C^−1^, indicating favorable stability of the dielectric constant with temperature in this range. However, as the temperature continues to rise, the dielectric constant–temperature coefficient exhibits a significant increase. 

In addition, the dielectric properties of PEN and PEN-based nanocomposite films with changing frequency at different temperatures are shown in Figure 12d–i. It can be clearly seen from Figure 12d–f that the dielectric constant of all PEN and PEN-based nanocomposites gradually increases with the increase in temperature. This is mainly due to the fact that the interface polarization gradually strengthens with the increase in temperature, and the molecular movement is improved, resulting in an increase in the dielectric constant. However, the dielectric constant of all films increases relatively slightly before 160 °C. This is because the movement of the molecular chains of PEN in this temperature range is limited, so that its dielectric constant remains relatively stable, which is consistent with the results of the dielectric constant–temperature coefficient in Figure 12c. Owing to the same reason, the dielectric loss of PEN and PEN-based nanocomposite films with changing frequency at different temperatures shows a trend similar to that of the dielectric constant, which is shown in Figure 12g–i. These findings confirm the outstanding dielectric properties–temperature stability of the PEN nanocomposite films up to 160 °C, making them suitable for elevated environments.

Moreover, Table 1 presents the dielectric properties of various composites filled with BN or TiO_2_ nanofillers. It is evident that many other polymer-based composite materials reported exhibit low dielectric constants and operate within temperatures below 160 °C. In addition, some PEN-based nanocomposites with similar inorganic functional fillers, such as PEN/CuPc-BN, PEN/TiO_2_, PEN/TiO_2_-CN, are listed in Table 1. Although these nanocomposites also have high operating temperatures, their dielectric constant is relatively low. Conversely, the PEN/(BN-TiO_2_)@(PDA + PEI) nanocomposites developed in this work have relatively high dielectric properties and can withstand temperatures as high as 160 °C, providing a significant advantage for their application in high-temperature environments. This characteristic ensures their suitability for application in demanding high-temperature conditions.

**Figure 13:**
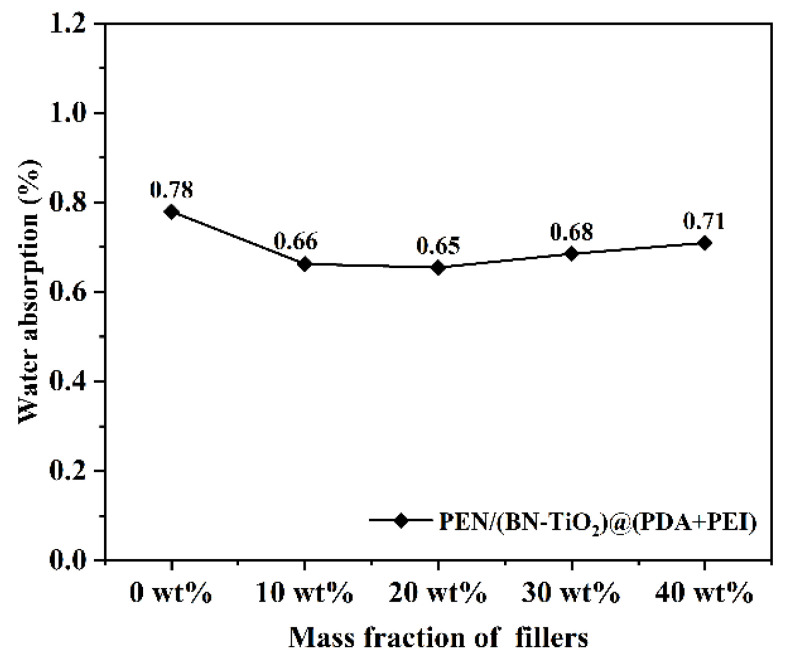
Water absorption of nanocomposite films.

The water absorption of nanocomposite films in different proportions is displayed in Figure 13. It can be seen that the water absorption of all nanocomposite films is below 0.8%, which is mainly attributed to the presence of a large number of hydrophobic nitrile groups and a benzene ring in the PEN molecular chain, resulting in lower water absorption. Furthermore, the introduction of filler content results in a slight decrease in water absorption of the nanocomposite film. This decrease is primarily due to the introduction of inorganic filler, which occupies the free volume within the PEN molecular chain, further compressing the available space. However, with the increase in the content of nanofillers, local agglomeration inevitably occurs in the PEN matrix, resulting in the formation of defects such as some holes, increasing the infiltration of water and slightly increasing the water absorption of the nanocomposites. These results indicate the nanocomposite film’s excellent hydrophobicity, making it well-suited for applications in electronic devices.

## 4. Conclusions

In summary, this work presents a technique for the fabrication of PEN-based nanocomposite films with outstanding heat resistance for applications in high-temperature environments. A novel three-dimensionally structured nanohybrid with a rough surface was successfully fabricated by tightly attaching TiO_2_ nanoparticles to BN nanosheets using a PDA and PEI co-modification method. Thereby, the nanohybrids were added to the PEN matrix as reinforcing fillers, and the functionalized nanocomposites were prepared via a simple solution-casting method. The results show that the properties of PEN nanocomposite films can be significantly improved by the introduction of (BN-TiO_2_)@(PDA + PEI) nanohybrids with three-dimensional structures. In particular, the *T*_g_ and the dielectric constant at 1 kHz of the nanocomposite films were increased by 3.9% and 162% at 40 wt% filler loading, respectively, results that are far higher than those of PEN. More importantly, it is observed that the dielectric constant–temperature coefficient of the nanocomposite films remains below 5.1 × 10^−4^ °C^−1^ over the temperature range of 25–160 °C, which indicates the excellent thermal stability of the PEN nanocomposites. It is believed that these nanocomposites have potential prospects in the field of high-temperature-resistant electronics.

## Figures and Tables

**Table 1:** Comparison of dielectric properties of different composite dielectric materials.

Samples	Content	Dielectric Constant (1 kHz)	Dielectric Loss (1 kHz)	Working Temperature (°C)	Ref.
RCH-BN	10%	6.78	~0.018	-	[44]
Polyester/surface-modified hBN	10%	~3.70	-	~100	[45]
Epoxy/h-BN	10%	~5.60	~0.090	<146	[46]
PPER/h-BN@SiO_2_	20%	~3.57	~0.006	-	[47]
LDPE/tr-TiO_2_	3%	~2.28	~0.0005	-	[48]
EP/S-TiO_2_	5%	~3.70	~0.018	-	[49]
PVA/TiO_2_	5%	3.55	-	-	[50]
PEN/BNNS	5%	~3.60	~0.020	~160	[51]
PEN/P@B PEN/CuPc-BN	10% 10%	~4.50 ~5.00	~0.010 ~0.013	<180 -	[52] [53]
PEN/TiO_2_	10%	~4.70	~0.013	<180	[54]
PEN/TiO_2_-CN	10%	4.02	~0.010	<180	[55]
PEN/(BN-TiO_2_)@(PDA + PEI)	10%	~5.20	~0.015	~160	This work

## Data Availability

Data are contained within the article.
